# Phenotypic plasticity of a generalist fish species resident to lotic environments: Insights from the Great Lakes region

**DOI:** 10.1002/ece3.10715

**Published:** 2023-11-20

**Authors:** Claire Hetzel, Patrick Forsythe

**Affiliations:** ^1^ Department of Natural and Applied Sciences University of Wisconsin‐Green Bay Green Bay Wisconsin USA

**Keywords:** biogeography, ecomorphology, geometric morphometrics, phenotypic plasticity, *Semotilus atromaculatus*, tributaries

## Abstract

Fish morphology is incredibly plastic and local/resident morphology can be influenced by factors including habitat, predation, resource availability, and water velocity. Through analysis of body shape using geometric morphometrics, we describe the degree of phenotypic plasticity within a generalist fish species resident to low‐order tributaries of Green Bay and Lake Michigan. We predicted that isolated populations of creek chub (*Semotilus atromaculatus*) would display plastic responses in body shape due to differences in selective pressures imposed by stream environments. We show that body shape of creek chub was significantly different between streams which are considered to be isolated populations, and while we expected body shape variation to remain constant between study years, we found that shape was not fixed and changed over time in the same manner among focal streams. The diversity of creek chub diet and degree of agricultural land use in the watershed were significant predictors of body morphology. The effect of resource availability and land use within the watershed demonstrates how selective pressures influence phenotypes at the population level. Our study provides insight into morphological changes of stream fish populations, which may be important in the context of changing ecosystems and novel conditions.

## Graphical abstract

Through analysis of body shape using geometric morphometrics, we describe the degree of phenotypic plasticity within a generalist fish species resident to low‐order tributaries of Green Bay and Lake Michigan. We show that body shape of creek chubs was significantly different between streams which are considered to be isolated populations, and while we expected body shape variation to remain constant between study years, we found that shape was not fixed and changed over time in the same manner among focal streams. The study provides insight into morphological changes of stream fish populations, which may be important in the context of changing ecosystems and novel conditions.

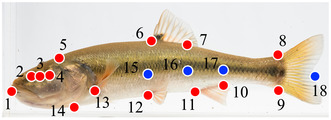

## 1 INTRODUCTION

Phenotypic plasticity is the expression of character traits produced by a single gene (West‐Eberhard, 1989), which allows individuals to maximize fitness across environmental conditions (Schlichting, 1986). Plasticity allows populations to respond to selective pressures in heterogenous environments (Via et al., 1995). Contemporary evolution is controlled by phenotypic plasticity (Callahan et al., 1997), and morphological aspects such as body shape exhibit plastic responses to selective pressures from sources including food availability, predation, and water velocity (Kelley et al., 2017; Langerhans et al., 2004; Olsson & Eklöv, 2005). Akin and Geheber (2020) show that morphological changes can occur in as little as 60 days while others have shown generational changes established in a population after 10–20 years (Cureton & Broughton, 2014).

Fish resident to streams and rivers face selective pressures from flow, predators, habitat modification, and food availability and all play a role in triggering morphologic changes (Langerhans et al., 2004; Lostrom et al., 2015; Scharnweber, 2020). Research to date has generally focused on comparing a single fish species between lotic and lentic habitats (Franssen, 2011; Haas et al., 2010; Lang et al., 2018; Ramler et al., 2016) showing that lake or reservoir populations are deeper bodied while stream populations are streamlined (Franssen, 2011; Haas et al., 2010). Langerhans (2008) suggests that river environments favor a streamlined shape to reduce drag while a deeper body shape is effective in low flow conditions to aid in burst swimming. Fewer studies have analyzed morphometric plasticity of fish populations among isolated streams but plastic responses have been reported as a function of increased urbanization and marked differences in stream flow (Kern & Langerhans, 2018). Generalist species benefit from adaptive plasticity to aid habitat colonization and to persist through environmental change (Griffiths, 2010; Poff & Allan, 1995).

Northeast Wisconsin provides a unique freshwater landscape centered on Green Bay, Western Lake Michigan and the tributaries that feed them. Total yearly precipitation in Wisconsin has increased 50–100 mm since 1950 (Dauwalter & Mitro, 2019), resulting in increased base flow variability. Fish are exposed to different potential selective pressures including open water predators, seiches, and temperature variation from north to south. Streams in Northeast Wisconsin also flow through a complex geology including the Niagara Escarpment which plays a role in shaping fish diversity and contributing to significant differences in the fish assemblages observed among streams (Allan, 2004; Koosman, 2015; McReynolds, 2020; Nau, 2019). The diversity of streams in this region, along with the influence of land use activities within the watershed and targeted efforts for restoration, presents an opportunity for a generalist species to display variable plastic responses, but such responses have never been described. We expect fish populations resident to different stream types (forested, developed, agricultural, wetland/grassland) to display morphological differences in response to variable in‐stream conditions.

Any number of species resident to Northeast Wisconsin tributaries could be used to explore phenotypic plasticity, but creek chub (*Semotilus atromaculatus*) are an excellent model species as they tolerate a wide range of stream conditions (Copes, 1978; Dinsmore, 1962). McMahon (1982) describes the creek chub as preferring to occupy clear water streams but tolerant of lower quality habitats, allowing their distribution in North America to extend east of the Rocky Mountains and south of Manitoba. Creek chub occupy stream patches with vegetation and woody debris and exhibit frequent, short‐distance movement among patches (Belica & Rahel, 2008). Known as one of the most abundant stream fish species, creek chub are opportunistic feeders and serve as important predators in low order streams but become prey sources when larger piscivores are present (Fitzgerald et al., 1999). Previous research has shown that creek chub in rural streams were more streamlined than those in urban streams, which was attributed to water velocity differences and populations adjusting their swimming mechanics (Kern & Langerhans, 2018).

We aim to describe the range of plastic responses of a ubiquitous stream resident fish and determine if observed shape variation can be explained by environmental drivers beyond river discharge. We expect that creek chub exhibit plasticity in body shape as environmental variation selects for generalist species (Griffiths, 2010). Specifically, we expect streams with marked differences in land cover, including the predominance of agriculture, to exhibit divergent body shapes, since agricultural land use has been shown to alter stream flow (Slater & Villarini, 2017) and thus drive a change in swimming mechanics (Langerhans, 2008). Second, we examined interannual variation in phenotypic plasticity and determine whether plasticity occurs over a short temporal scale. To our knowledge, no study to date has tested whether plastic morphological patterns are stable on relatively short time scales, such as over one calendar year. Exploring geographically separated creek chub populations will allow us to describe the range of body shape plasticity of this generalist species.

## 2 MATERIALS AND METHODS

### 2.1 Study area

**FIGURE 1:**
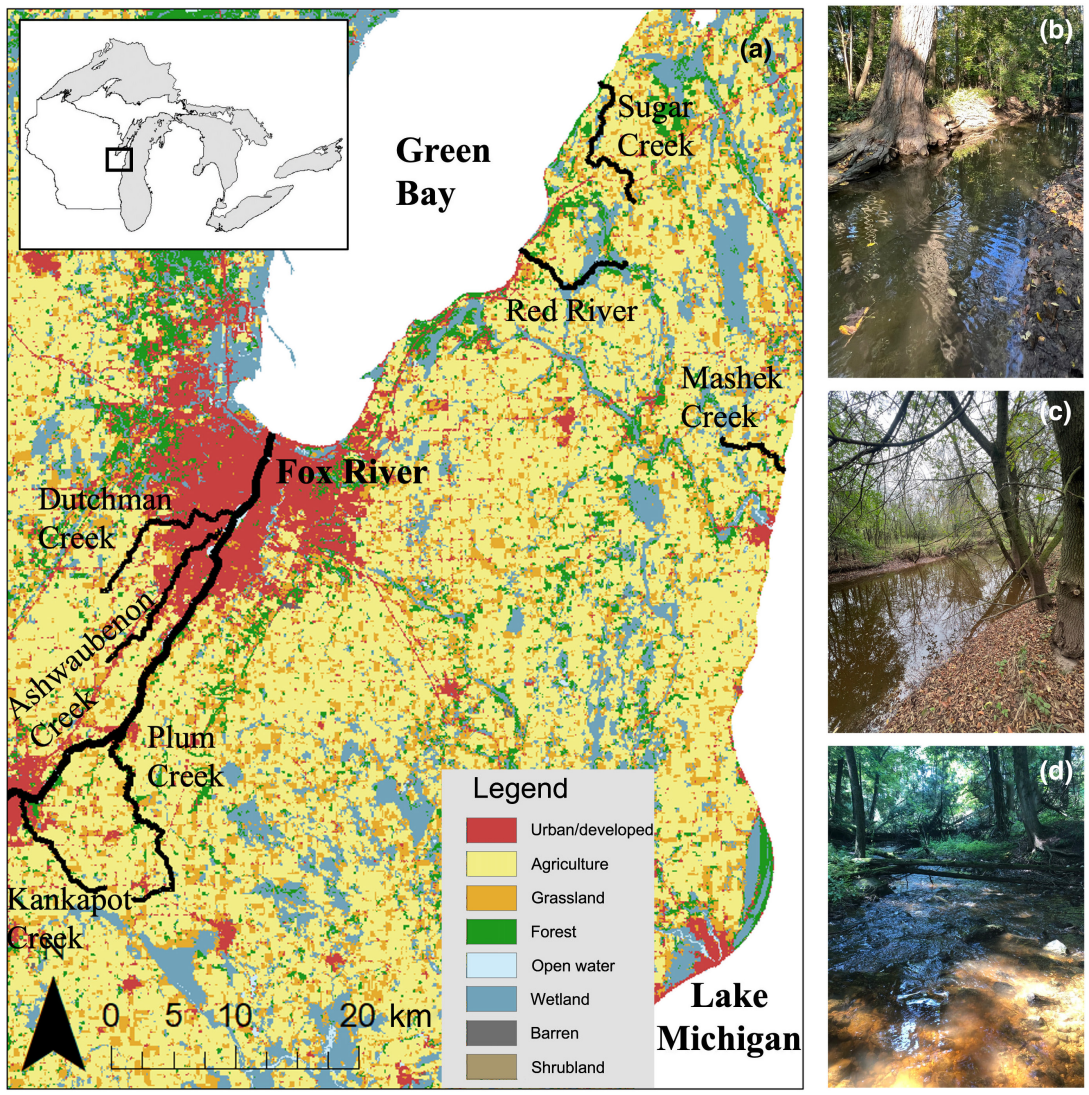
Geographic locations of (a) the seven stream sites sampled in Northeast Wisconsin in relation to the Great Lakes system, with select site photos that illustrate the three main waterbodies in the region: (b) Sugar Creek, which empties into Green Bay; (c) Ashwaubenon Creek, which empties to the Fox River; and (d) Mashek Creek, which empties into Lake Michigan.

Seven streams were selected based on their proximity to Green Bay, Wisconsin, their geographic separation, and the relative degree of creek chub dominance (Nau, 2019). Sugar Creek and Red River, the two northern‐most sites, flow directly into Green Bay while Ashwaubenon, Dutchman, Kankapot, and Plum Creeks flow into the Fox River, which confluences with the Green Bay, and Mashek Creek is located on the eastern side of the study region and flows into Lake Michigan (Figure 1). Plum Creek is surrounded by farmland and has very few riffles while Kankapot Creek has step streambanks and a bedrock streambed throughout the stream length. Ashwaubenon and Dutchman Creeks are located within urban areas and the Ashwaubenon Creek site has few riffles and pools, while the Dutchman Creek site had deep pools created by downed logs with high surrounding streambanks. Both Red River and Sugar Creek flow through a forested landscape with trees and shrubs along the streambanks and are more isolated from urban areas. Mashek Creek has many downed logs within the stream which tends to create significant pooling throughout the reach. Altogether, our focal study streams represented a north–south gradient predicted to have an influence on their physical and biological features and potentially the plastic responses of the resident fish. Six streams were sampled in September through October 2020 and four streams were sampled in September through October 2021 (Table 1). Three sites including Red River, Sugar Creek, and Ashwaubenon Creek were resampled in 2021.

**TABLE 1:** Site descriptions and locations of stream sampling targeting creek chub for geometric morphometric analysis; includes the abiotic site characteristics of watershed size, land cover composition, and average temperature during the sampling period.

Site	Year	Month	Lat (N)	Long (W)	Watershed size (km^2^)	% Agriculture land cover	% Developed land cover	Average temperature (°C)
Ashwaubenon Creek	2020 2021	October	44.4133	−88.1268	182.40	54.09	26.45	18.46
Dutchman Creek	2020	October	44.4830	−88.0869	182.40	54.09	26.45	18.98
Kankapot Creek	2020	September	44.2755	−88.2680	135.25	52.68	24.32	NA
Mashek Creek	2021	September	44.50265	−87.48652	218.19	49.83	4.45	18.86
Plum Creek	2020	September	44.3054	−88.1713	135.25	52.68	24.32	19.88
Red River	2020 2021	September	44.6676	−87.7445	223.96	38.69	6.65	19.32
Sugar Creek	2020 2021	September	44.7871	−87.6594	223.96	38.69	6.65	18.73

*Note*: Temperature values are reported from 2020 only, except for Mashek Creek which was only sampled in 2021.

Watershed data was gathered from the Wisconsin Department of Natural Resources 2018 (DNR) and land cover data was extracted from the Wiscland 2.0 GIS dataset via the Wisconsin DNR GIS Open Data Portal and is reported as a percent of the total land cover in the watershed. Watershed area and land cover composition are provided in Table 1. On average, total watershed size among our focal tributaries was 185.9 km^2^. Agricultural land cover was 48.7% with developed land cover 17.0% of the total land cover. Plum Creek and Kankapot Creek watersheds were dominated by agricultural practices with a quarter of the watershed consisting of developed land and a small amount of grassland. Ashwaubenon Creek and Dutchman Creek watershed land cover is almost identical to that of Plum and Kankapot Creeks, though the total watershed area is 47.15 km^2^ larger. Conversely, Red River and Sugar Creek watershed consisted of agriculture, grassland, and wetland land cover types. Mashek Creek has the smallest percent of developed land within the watershed, and is comprised mainly of agricultural (40%), grassland (20%), and wetland (20%) land cover types. The United States Geological Survey (USGS) maintains water gauges in three of our study streams including Ashwaubenon Creek, Dutchman Creek, and Plum Creek. We report discharge data from these streams to supplement field data.

### 2.2 Field sampling

Temperature loggers were placed in study streams roughly 1 month prior to the expected sampling date and set to log water temperature hourly. Fish sampling was conducted via single‐pass backpack electrofishing with a pulsed DC current at 75 volts using Model AS2 Backpack Electrofisher from Aqua Shock Solutions. Bergman et al. (2011) determined that single‐pass stream electroshocking is a sufficient method for sampling Wisconsin streams. Two backpack units were used in a side‐by‐side fashion working from downstream to upstream, followed by additional netters and spotters to capture all fish. Two backpack operators allow for maximal capture of individuals without increasing reach length (Kimmel & Argent, 2006). Reach length varied by site, though we aimed to sample a 100–300‐m reach until a sufficient creek chub population was collected following Wisconsin DNR protocols (Guidelines for Assessing Fish Communities of Wadeable Streams in Wisconsin v2.0). Streams were visited once per week for two consecutive weeks to ensure the population was sampled comprehensively. All fish were identified to species, counted, and non‐creek chub species were returned to the stream. To estimate the fish community diversity, Simpson's Index of Diversity was calculated (1 − *D*), which emphasizes species evenness and common species (Troast et al., 2020):

(1) $$D=\frac{\sum n\left(n-1\right)}{N\left(N-1\right)}$$

Creek chub dominance was calculated for each site as the percent of creek chub individuals out of the total numbers of fish collected:

(2) $$\frac{\#\text{Of creek chub}}{\text{Total}\#\text{of individuals present}}\times 100$$

Creek chub were transported to the lab and held in medium‐sized tanks for no longer than 2 days and housed in water collected from their native stream to reduce stress and mortality, until photographing took place.

### 2.3 Gut content analysis

Immediately following field sampling, a subset of our creek chub collection was put on ice, then stored in a freezer for later gut content analysis. We aimed to collect between 5 and 10 individuals per stream for gut content analysis (Table 2). Fish were thawed and the entire digestive tract was removed and rinsed with DI water. The internal viscera was removed from the digestive tract and the tract was measured according to length and weight (Quist et al., 2006). The contents were expelled, and prey items were identified to the lowest taxonomic unit possible and preserved in 70% ethanol. Diet categories were established to include macroinvertebrate order, terrestrial insects, plant material and detritus, unidentified material, and parasites. Parasites were not identified and are grouped together in a general parasite category. Figure S3 provides specimen photos of documented parasites.

**TABLE 2:** Number of creek chub per site used in geometric morphometrics (GM) and gut content analysis (GCA), with the average and range of fish total length collected during two sampling seasons in 2020 and 2021.

Site	Total *n*	Length range (mm)	Average length (mm)	Fall 2020			Fall 2021		
				*n* (GM)	*n* (GCA)	Length range (mm)	*n* (GM)	*n* (GCA)	Length range (mm)
Sugar Creek	112	37–232	115	35	10	37–204 (116)	51	8	39–225 (124)
Red River	80	38–235	126	35	14	38–235 (108)	33	10	54–219 (138)
Plum Creek	164	52–212	94	76	16	62–212 (100)			
Kankapot Creek	288	30–211	79	52	15	43–204 (101)			
Ashwaubenon Creek	99	76–209	130	42	5	76–208 (125)	26	6	85–209 (154)
Dutchman Creek	90	50–192	96	45	4	53–192 (108)			
Mashek Creek	74	45–182	100				74	9	45–182 (100)

*Note*: Total *n* and average length include all creek chub collected during both sampling seasons (individuals not included in morphometric and diet analysis were released back to the stream).

The frequency of occurrence (% *F*), numeric abundance (% *N*), and volumetric point method (% *V*) (Hynes, 1950; Zacharia, 2017) were computed and combined for the index of relative importance (IRI) (Hyslop, 1980):

(3) $${\mathrm{IRI}}_{i}=\left(\%N_{i}+\%V_{i}\right)\times \%F_{i}$$

The diversity of prey items was calculated using Simpson's Diversity Index to estimate the diversity of diet for creek chub separately according to population (Tobler, 2008), where *n* is the total number of organisms of a species, and *N* is the total number of organisms of all species:

(4) $$D=\frac{\sum n\left(n-1\right)}{N\left(N-1\right)}$$

### 2.4 Geometric morphometrics

**FIGURE 2:**
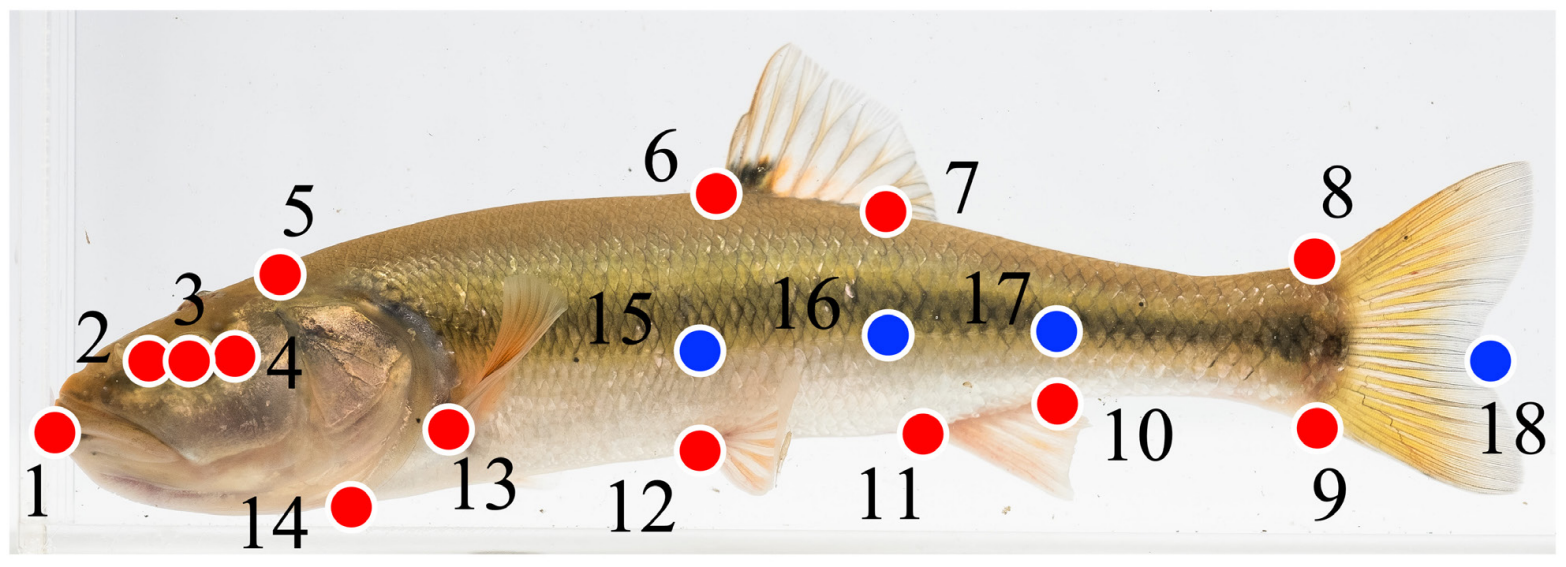
Position of the landmarks used in the geometric morphometric analysis. Homologous landmarks (red circles) include: (1) anterior tip of snout on upper lip; (2) anterior margin of eye; (3) center of eye; (4) posterior margin of eye; (5) posterior base of skull; (6) anterior insertion of dorsal fin; (7) posterior insertion of dorsal fin; (8) dorsal insertion of caudal fin; (9) ventral insertion of caudal fin; (10) posterior insertion of anal fin; (11) anterior insertion of anal fin; (12) origin of pelvic fin; (13) origin of pectoral fin; (14) ventral insertion of operculum. Four semi‐landmarks (blue circles) were used for unbending, including: (15) lateral line above LM 12; (16) lateral line below LM 7; (17) lateral line above LM 10; (18) fork of caudal fin.

Landmark‐based geometric morphometrics techniques were used to obtain shape data for individual fish (Adams et al., 2013; Bookstein, 1996; Rohlf & Marcus, 1993; Rohlf & Slice, 1990). Creek chub were placed in photoboxes and all specimens were photographed alive on the left lateral side using a Nikon COOLPIX L310 digital camera placed approximately 35 cm away from the photobox. Live specimens provide an opportunity to minimize preservation effects on specimens by ethanol or formalin, which Martinez et al. (2013) have shown to exaggerate the variation among specimens. Other studies have utilized this approach of photographing live specimens, which we aimed to replicate (Kelley et al., 2017; Langerhans et al., 2003; Lostrom et al., 2015). All photographs included a scale bar to standardize all landmarks. Images were digitized using ImageJ v1.53a (https://imagej.nih.gov/ij/) and 18 total landmarks were placed (Figure 2), corresponding to 14 homologous structures and 4 semi‐landmarks along the lateral line to unbend specimens (Armbruster, 2012; Haas, 2011; Kern & Langerhans, 2018). The unbending procedure was carried out using tpsUtil (Rohlf, 2016) to remove the variation caused by natural curvature through the caudal fin that was present in live specimens (Haas, 2011). Landmarks 15–18 were omitted from morphological tests after all specimens were unbent (Appendix S1). Two separate analyses were performed. First, a comparison between the seven streams using specimens collected in the first sampling event for each stream, and second, a comparison between the 2 years for the three repeated sites.

Using MorphoJ v1.07a, all landmarks were scaled to the same size and a Generalized Procrustes analysis was completed to generate the mean shape of all individuals by removing size and position, the extraneous variables of shape (Klingenberg, 2011). We regressed Procrustes coordinates against centroid size, which is the square root of the sum of squared distances of all landmarks from an object's centroid, to remove allometric effects and used the regression residuals in subsequent analyses (Klingenberg, 2011; Scharnweber, 2020). A canonical variate analysis (CVA) was conducted to determine the significance of shape variation between sites followed by a discriminant function analysis (DFA) to visualize the shape change between pairs of sites (Scharnweber, 2020). These preliminary statistical analyses were completed using Morpho J v1.07a (Klingenberg, 2011).

### 2.5 Modeling body shape and environmental factors

Relative warp scores (RWs) were generated using tpsRelw (Rohlf, 2015) and used to represent shape as a variable in modeling. Each relative warp is a direction of shape change from the mean shape of a sample and represents variation from the mean (Rohlf & Marcus, 1993). We included only relative warps that represented at least 5% of the total variation. We ran a multiple linear regression with shape (using 6 RWs that explained 70.46% of the total variation) as the dependent variable with size (centroid size), stream temperature (°C), watershed size (km^2^), land cover (as % developed and % agriculture), fish diversity, creek chub dominance, and diet diversity as independent variables (Beachum et al., 2016; Kern & Langerhans, 2018). Correlation between predictors was tested and the best model was selected with the lowest Akaike's information criterion (AICc) value (Haas et al., 2015). Statistical modeling was performed in R using the *car* package (R Core Team, 2021).

## 3 RESULTS

### 3.1 Stream conditions

Notable differences occurred between the two sampling years with respect to rainfall and stream morphology. Changes in pool location and depth, which are essential creek chub habitat (McMahon, 1982), were especially obvious in Red River which tends to be influenced by the highly variable internal seiche effects common to the Green Bay system (Gottlieb et al., 1990). Stream temperature was not widely different among streams nor as a function of geographic location as we expected (Figure S1). Stream discharge for the three focal streams with dedicated gauging stations (Table S1) reveals the flashy nature of the focal streams, as they increased sporadically above the average baseflow. These differences in discharge are largely due to precipitation events, which are evident in Figure S2.

### 3.2 Gut content analysis

**FIGURE 3:**
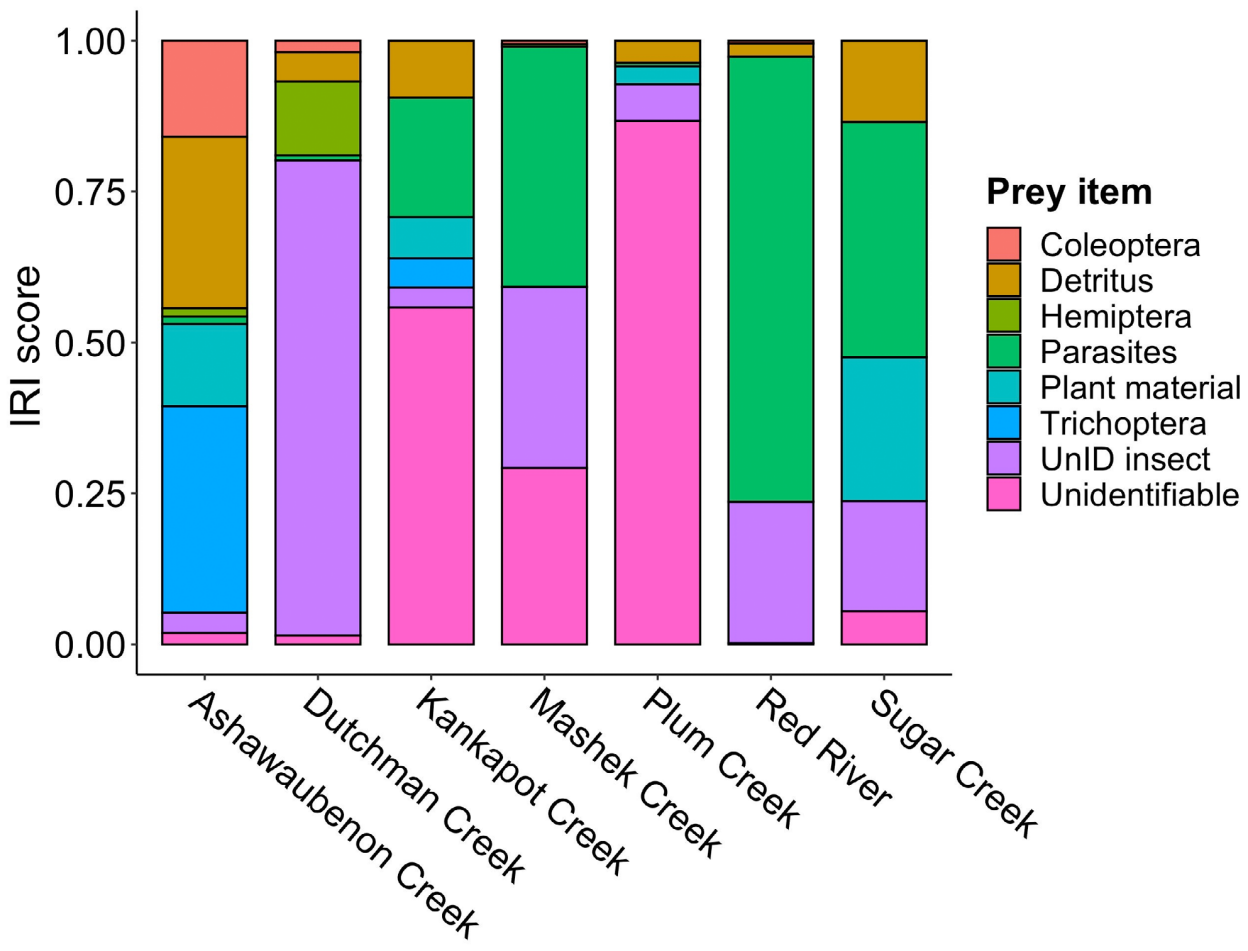
Relative importance (%IRI) of the most dominant prey items for each creek chub population, as revealed by gut content analysis. Prey items include those that accounted for at least 10% relative importance for at least one population. See Table S2 for complete IRI scores with all prey categories.

Twelve creek chub (12.4%) had empty stomachs, while the remaining 87.6% contained some food items (Figure 3). Streams with a higher percentage of agricultural land use in the watershed (Dutchman Creek and Plum Creek) had high amounts of unidentifiable prey items. The more urban systems had the highest IRI values for Hempitera, Trichoptera, Coleoptera, and detritus (%IRI = 11.2, 33.6, 15.6, and 27.9, respectively). Streams characterized by forested land cover had the highest percentage of plant material (%IRI = 23.8). Empty stomachs were found in 11 individuals from agricultural sites, and only one individual from the forested sites. Streams with the highest prey diversity, as quantified by Simpson's diversity index (Tobler, 2008), did not follow land use patterns, but include Kankapot Creek, Dutchman Creek, and Mashek Creek (1 − *D* > 0.75), while Red River had the lowest prey diversity (1 − *D* = 0.422). The forested streams all had high IRI scores for parasites (%IRI = 72.0, 38.9, and 34.9; Table 3).

**TABLE 3:** Biological characteristics for each site, including average relative warp scores (RW1–RW6), average centroid size, creek chub dominance (as percent of total individuals at each site), and fish community and prey diversity (as Simpson's Diversity Index).

	Ashwaubenon Creek	Dutchman Creek	Kankapot Creek	Mashek Creek	Plum Creek	Red River	Sugar Creek
Centroid size	3.45	3.42	3.45	3.43	3.423	3.44	3.42
RW1	0.0053	−0.0039	−0.0058	−0.0093	0.0078	−0.0054	−0.0043
RW2	0.0003	−0.0050	−0.0001	−0.0028	−0.0012	0.0069	0.0020
RW3	0.0007	0.0015	−0.0064	−0.0015	0.0028	−0.0012	0.0018
RW4	−0.0005	−0.0019	−0.0006	−0.0048	0.0016	0.0022	−0.0017
RW5	−0.0019	0.0015	0.0017	0.0015	−0.0017	0.0013	0.0003
RW6	0.0008	0.0021	−0.0022	−0.0012	−0.0015	0.0008	0.0019
Fish diversity	0.67	0.78	0.50	0.68	0.59	0.83	0.80
% Creek chub	56.76	44.56	69.33	51.39	58.46	29.66	38.04
Prey diversity	0.67	0.78	0.83	0.79	0.62	0.42	0.74

### 3.3 Comparative morphometrics

**FIGURE 4:**
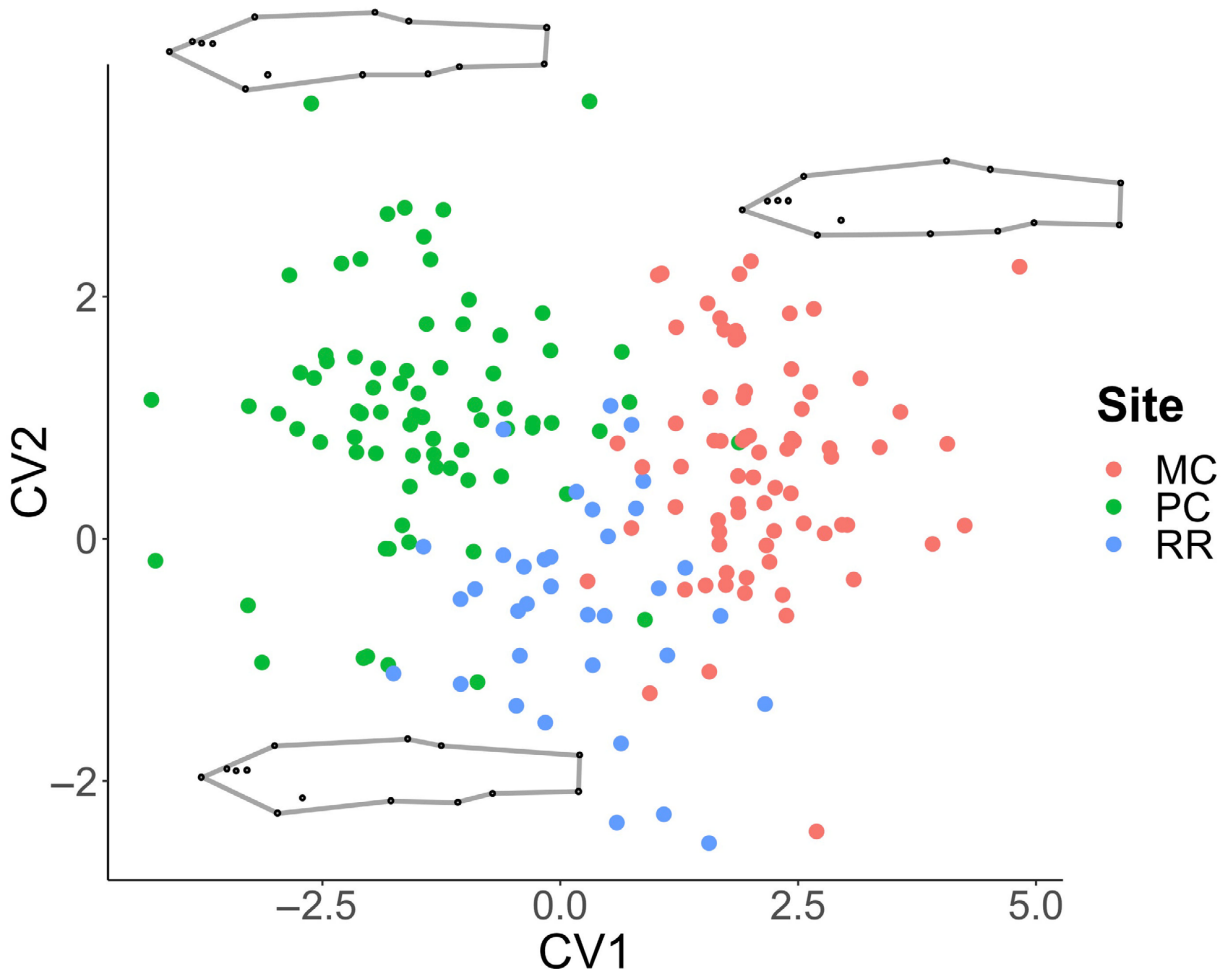
Canonical variate analysis plot of the three stream populations that had a body shape different from all other streams: Plum Creek (PC), Mashek Creek (MC), and Red River (RR) populations. Wireframes represent the average shape for each population, magnified by 3 to highlight shape differences.

**FIGURE 5:**
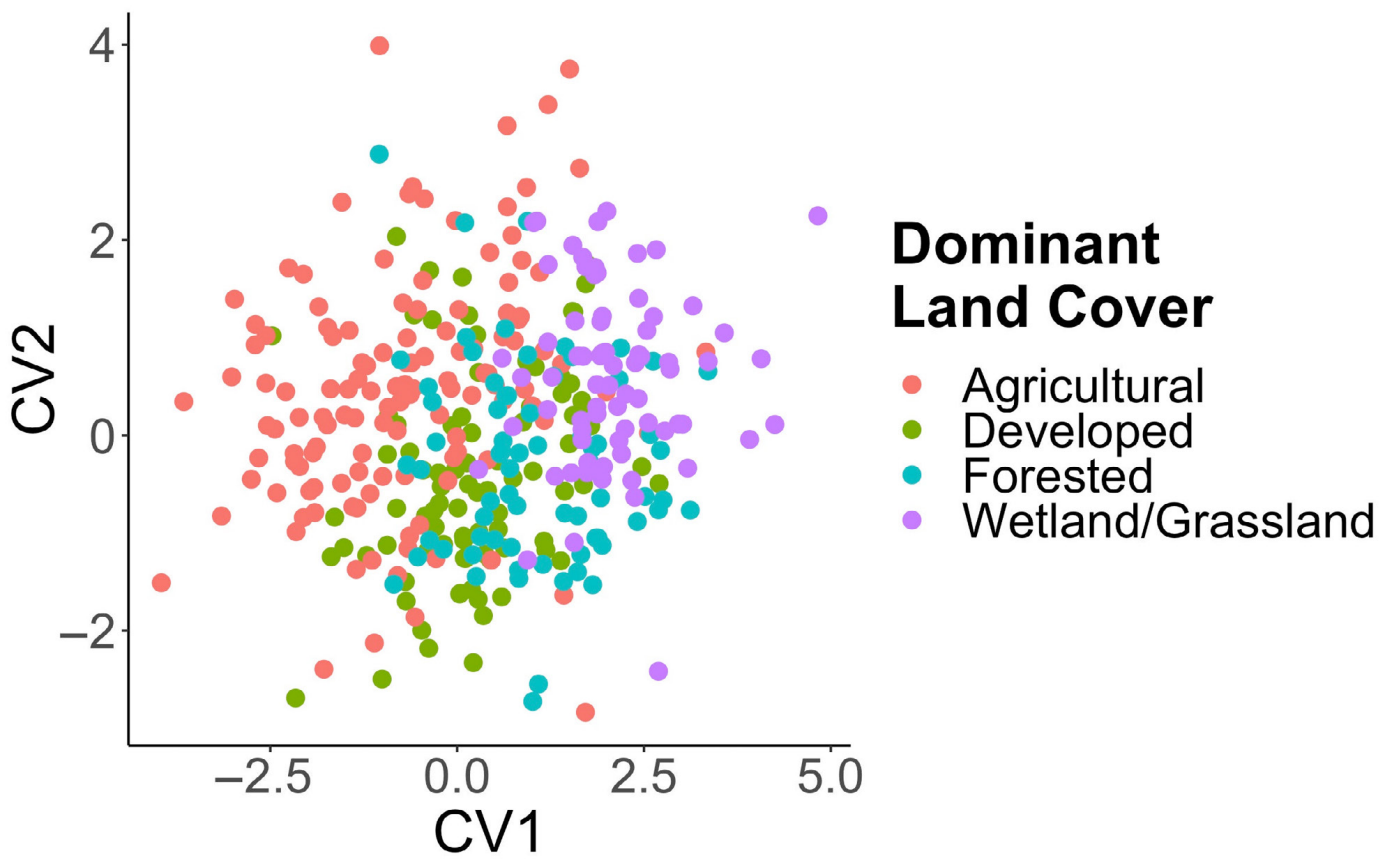
Canonical variate analysis of all creek chub populations grouped according to dominant land cover type. The categories included are agricultural (Plum Creek and Kankapot Creek), developed (Ashwaubenon Creek and Dutchman Creek), forested (Sugar Creek and Red River), and wetland/grassland (Mashek Creek).

Canonical variate analysis revealed body shape differences between creek chub populations (Table 4), with the Mashek Creek, Plum Creek, and Red River populations accounting for the greatest morphological differences between sites. Fish shape of the Mashek Creek population was significantly different from all other populations (*p* < .0001) and had the deepest midsection, in relation to the width of landmarks 6–7 and 10–11. Plum Creek and Red River creek chub body shape was significantly different from five and four other populations, respectively (Table 4, Figure 4). The first CV axis accounts for 52.7% of the total variation and represents a creek chub shape that is deeper bodies through the midsection with a slimmer head shape than the consensus shape (full CV plot provided in Figure S4). The second CV axis represents 19.5% of the total shape variation and indicates a body shape that is streamlined with a larger head size than the consensus shape. Mashek Creek fish have a more upturned mouth than fish from Plum Creek and Red River. The paired stream locations did not correspond in terms of general shape (streamlined or deep‐bodied) as only the Ashwaubenon Creek and Dutchman Creek populations were both streamlined, while the paired Plum and Kankapot Creeks and the Red River and Sugar Creek pair exhibited opposite general body shape (Figure S5). Fish were different morphometrically based on dominant land use categories, especially comparing the agricultural and wetland/grassland streams (Figure 5), which are separated out in morphospace across the first CV axis.

**TABLE 4:** Results of Canonical Variate Analysis between body shapes of all six streams, with each pairwise relationship described.

	AC		DC		KC		MC		PC		RR	
	*S*	*p*	*S*	*p*	*S*	*p*	*S*	*p*	*S*	*p*	*S*	*p*
DC	0.0119	.0036										
KC	**0.0152**	**<.0001**	0.0085	.0557								
MC	**0.0202**	**<.0001**	**0.0174**	**<.0001**	**0.0162**	**<.0001**						
PC	0.0085	.0504	**0.0151**	**<.0001**	**0.0166**	**<.0001**	**0.0223**	**<.0001**				
RR	**0.0153**	**.0004**	**0.0136**	**.0002**	0.0106	.0246	**0.0108**	**.0009**	**0.0170**	**<.0001**		
SC	0.0130	.0062	0.0093	.0863	0.0106	.0216	**0.0131**	**<.0001**	**0.0161**	**<.0001**	0.0083	.4217

*Note*: Bonferroni‐corrected *p‐*value (*p* = .0024) for 21 pairwise relationships. Procrustes distance (*S*) and *p*‐values, significant differences are shown in bold.

**FIGURE 6:**
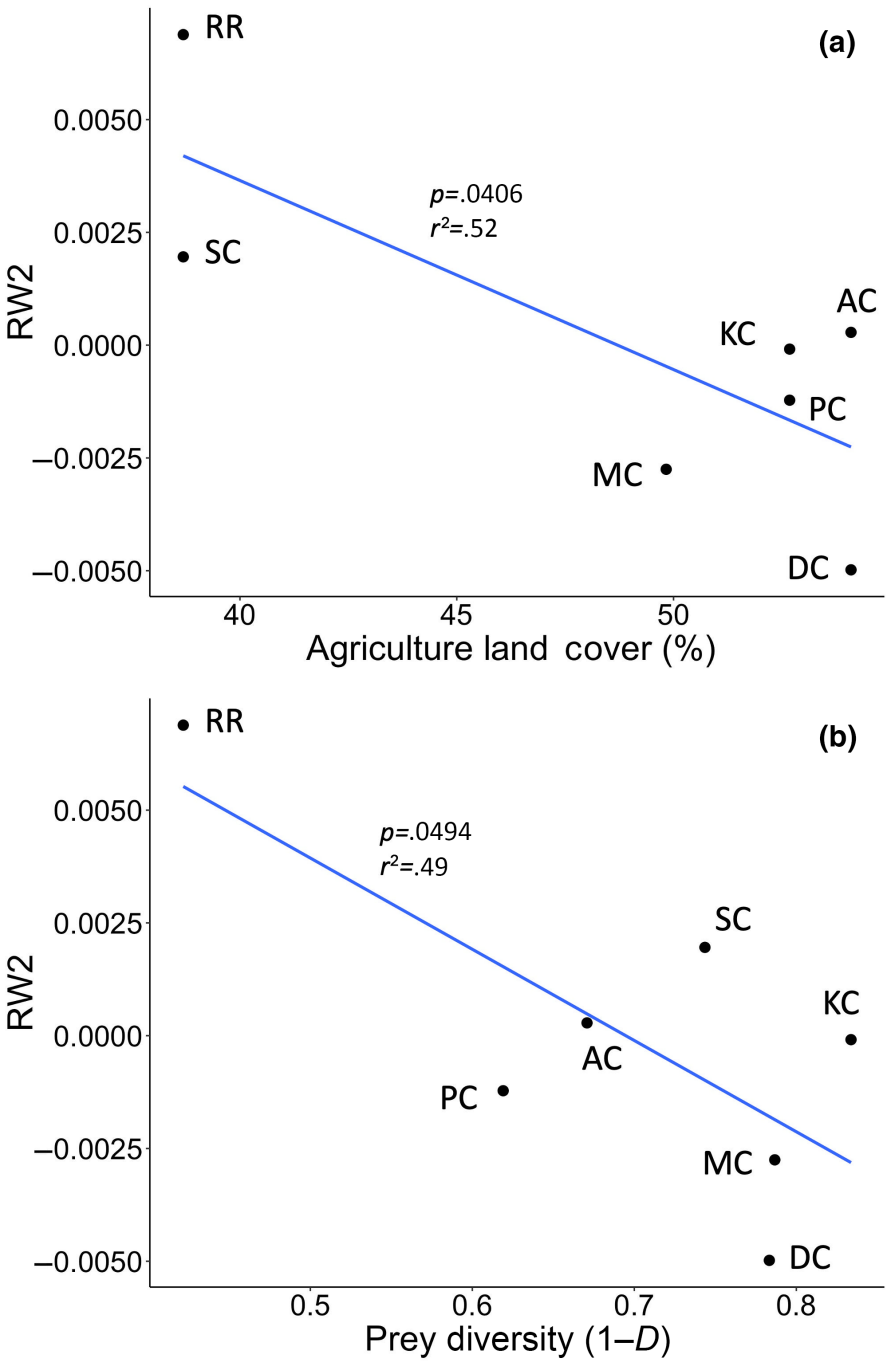
Relationships between fish body shape and significant predictors from best regression models including (a) RW2 and % agriculture land and (b) RW2 and prey diversity. Relative warp 2 represents a more streamlined shape with longer caudal fin.

Multivariate analysis of the second major axis of variation in body shape (RW2) revealed relationships with agricultural land cover and prey diversity, but that temperature and centroid size did not influence morphology (Table 5). Correlation between some of the predictor variables was relatively high (0.7–0.8) and these models were not explored in detail. Variables in the final model were not highly correlated. Relative warp analysis revealed the first six RW scores each accounted for at least 5% of the total variation and 70.46% of the variation cumulatively (Table 6). Only the first two RWs were retained in regression models due to the reduction in predictive power with subsequent relative warp scores. The second relative warp represents 16.3% of the total shape variation and features a streamlined body with a longer caudal fin and fin attachments shifted forward. Regression of RW2 and agricultural land cover indicates that as the amount of agricultural land use in the watershed size increases, fish shape becomes deeper through the dorsal fin and the eye position is higher (Figure 6a). Sugar Creek and Red River had the lowest amount of agriculture in the watershed and had positive RW2 scores, while the streams with the highest amount of agricultural land cover, Ashwaubenon and Dutchman Creeks, were further separated along RW2, where the Ashwaubenon Creek population was more streamlined than the Dutchman Creek population.

**TABLE 5:** MANOVA results for the first six relative warps (RW), with the best fitting model for each RW displayed.

Dependent variable	Model terms	*F*	df	*p*	*r* ^2^	AICc	△AICc
RW 1	% Developed land	3.08	1, 5	.1359	.2576	−71.43	36.24
RW 2	% Agriculture land + prey diversity	7.14	2, 4	**.0479**	.6718	−83.86	23.81

*Note*: Significant models are indicated where *p* is bold. See Table S3 for all model results.

**TABLE 6:** Relative warp scores (RW) and their corresponding percent of variation and cumulative percent.

RW	% Variance	Cumulative %
1	17.69	17.69
2	16.27	33.96
3	14.29	48.25
4	9.34	57.59
5	7.33	64.92
6	5.54	70.46

*Note*: Only warps accounting for at least 5% of variation are included.

Regressing RW2 with prey diversity of each population revealed a significant relationship (*p* = .0494) where increasing prey diversity results in negative RW2 scores (Figure 6b). Fish shape shifts toward a more streamlined average shape as prey diversity increases. The Red River and Dutchman Creek populations represent the lowest and highest prey diversity score, respectively, along with the most extreme RW2 scores. The Red River population is more streamlined through the dorsal region than the Dutchman Creek population, and the Dutchman Creek average shape has fin attachments positioned more posterior compared with the Red River population (Figure S5). At median levels of prey diversity (around 0.60), populations retain an average shape very close to that of RW2, with warp scores very close to zero, which represents a streamlined body shape and elongated caudal fin. Notably, two populations deviate the most from the consensus shape of RW2 in both models of agricultural land cover and prey diversity. In models for each variable, Red River has the largest warp score and is characterized by a longer head shape and slightly upturned mouth compared to the average RW2 shape, while Dutchman Creek has the lowest warp score and has fin attachments shifted posteriorly compared with RW2.

### 3.4 Interannual variation in shape

**FIGURE 7:**
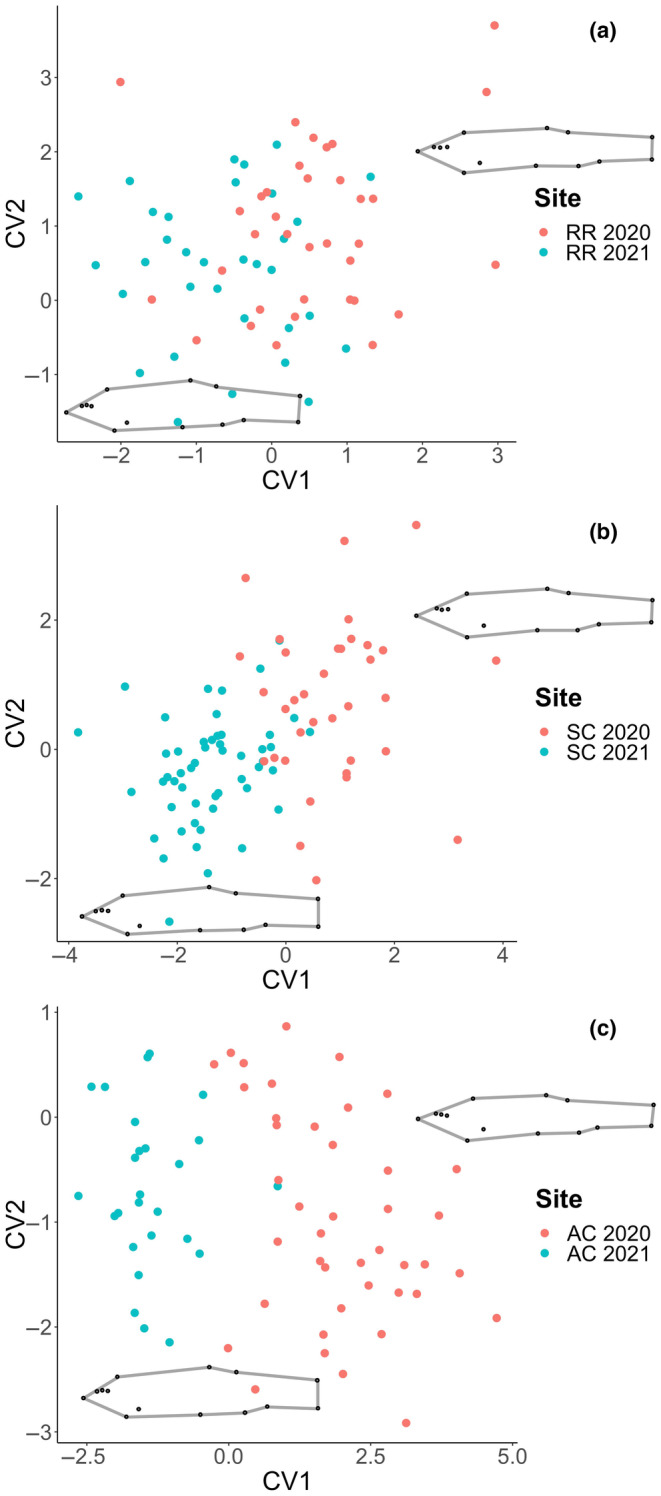
Morphological variation associated with CV1 and CV2 for the interannual comparison of each stream (a) Red River, (b) Sugar Creek, and (c) Ashwaubenon Creek. Wireframes are magnified by 3 to visualize body shape changes from 1 year to the next for each population.

Canonical variate analysis of populations from 2020 to 2021 resulted in significant relationships of body shape (*p* < .0001) among all three streams across the two sampling years (Table 7). Though we did not expect to observe differences in body shape between the same stream population from 1 year to the next, interannual differences in body shape of populations of the same stream were reported (Figure 7). When populations of the same stream were compared, projection into morphospace revealed separation between the two sampling years. The first CV axis accounts for more than half (57%) of the total shape variation between the two sampling years for each population and represents a streamlined body shape with an upturned mouth. For all three streams, the creek chub populations separated along the first CV axis and became deeper bodied in 2021 compared to 2020. The Ashwaubenon Creek populations exhibited the greatest shape change of the repeated sites, separating the most along CV1 in the 2021 sampling year. The Sugar Creek population remained the most streamlined of the three streams in 2021. The second CV axis explains 19.0% of the shape variation and is characterized by shifts in fin attachment placements and a narrower caudal fin. The three populations did not exhibit as much variation along the CV2 axis compared with CV1 from 2020 to 2021.

**TABLE 7:** Interannual comparison of fish body shape with canonical variate analysis.

	AC1		RR1		SC1	
	*S*	*p*	*S*	*p*	*S*	*p*
AC2	**0.0244**	**<.0001**				
RR2			**0.0135**	**.0017**		
SC2					**0.0132**	**<.0001**

*Note*: Bonferroni‐corrected *p*‐value (*p* = .0033) for 15 pairwise comparisons. Where AC1, R1, and S1 are the first sampling year (2020) and A2, R2, and S2 are the second sampling year (2021). Procrustes distance (*S*) and *p*‐values with significant differences shown in bold.

## 4 DISCUSSION

Morphometric differences between isolated populations of creek chub resident to streams in Northeast Wisconsin are primarily centered on overall body width and mouth shape. Fish shape was influenced by agricultural land cover and diet diversity, both of which induced a deeper midsection as these values increased. Preliminary work on geographically separated stream fish populations by Guill et al. (2000) showed both intra‐ and interspecific morphological variation of three darter species resident to the Southern United States but did not address environmental contributors to shape change. Beachum et al. (2016) documented stream fish shape changes as a function of stream flow, with increasing discharge resulting in species‐specific morphological changes across a wide range of geographic separation for two stream fish. The results presented here provided further evidence that isolated stream fish populations display divergent morphologies as a function of environmental pressures. Our analysis of interannual patterns of shape revealed that on as short of a timescale as 1 year, populations display plastic morphologies. Models examining relationships between fish shape and environmental variables identify agricultural land cover and prey diversity as the most influential factors in the study streams. In tandem, the stream comparison and interannual analysis demonstrate the complexity of selection pressures on a population and highlights the importance of adaptive phenotypic plasticity for a species' success under dynamic conditions.

Agricultural land cover was shown to induce phenotypic plasticity where fish resident to streams with greater agricultural land cover were more streamlined. A streamlined shape is more conducive to swimming mechanics in conditions with faster flow as it allows the fish to maximize thrust speed while reducing drag during steady swimming (Langerhans, 2008). This morphology may be useful in agricultural streams which have been shown to have enhanced flow speed due to decreased infiltration of precipitation in the watershed (Juckem et al., 2008). Few morphometric studies have included specific land cover types as a predictor of shape, or simply include levels of urbanization as a measure of stream flow. While Kern and Langerhans (2018) describe morphometric differences among rural and urban populations of creek chub, they did not distinguish agricultural land use from the rural stream category. In the Northeast Wisconsin study region where widespread agricultural use in the watershed is prevalent, it is especially relevant to consider how land use may affect physical stream characteristics beyond flow velocity. The current dataset is limited by the incomplete range of both agriculture land cover and prey diversity values (as illustrated by Figure 6). The study sites range from 39% to 52% agricultural cover and six out of the seven streams have a Simpson's diversity >0.60. The current study would benefit from streams with much less agricultural land cover to determine if there is indeed a breakpoint around 50% of agricultural land cover, where shape becomes more streamlined. The narrow ranges of both variables present a challenge to determine if the breakpoints along RW2 are simply a function of the data or are a true representation of the influence of these two variables.

Differences in discharge in the pre‐sampling period in 2020 compared with 2021 further demonstrate the variable conditions of these streams. Morphometric studies began describing stream flow by comparing lentic and lotic habitats but are moving toward comparing streams with variable flow speeds. Franssen 2013, Harris, et al. (2013) and Akin and Geheber (2020) show that stream discharge can induce phenotypic changes in fish shape and Rudolfsen et al. (2017) documented phenotypic changes in sculpin shape across a gradient of stream flow. While our data does not address this variable directly, measuring stream discharge in conjunction with testing the effects of land cover in the watershed could further illustrate the overall effects of land use on stream fish populations and their ability to adapt to variable conditions.

Creek chub resident to streams with high levels of diet diversity had a body morphology characterized by a shorter head shape with the tip of the snout positioned downward. This type of mouth shape indicates feeding behaviors centered on items lower in the water column or on the stream bottom, indicating resource polymorphism allowing for feeding lower within the water column. Morphometric differences in eye and mouth shape have been found to occur as a result of changes in diet due to resource partitioning (Skoglund et al., 2015). An altered head shape indicates possible changes in resource availability, which has been shown to change along with canopy cover and physical conditions of a stream (Kiffney et al., 2014), and reflects the influence of habitat complexity on fish adaptations. The Dutchman Creek and Mashek Creek populations illustrate this, as both streams are characterized by high levels of agricultural land cover and a high diet diversity, and both exhibit an average fish shape that is deeper through both the midsection and head area. Andersson et al. (2005) found habitat complexity to negatively affect the foraging ability of fish, which may be a contributing factor in the present study given the significant results with agricultural land use. Here, Red River has the least diverse diet, but we do not consider it to have low habitat complexity overall. The land cover in the surrounding watershed is made up of agricultural, wetland, grassland, forest, and urban influences, indicating potentially high habitat complexity. We also observed physical changes in stream structure between 2020 and 2021 at the Red River site, which suggests that habitat complexity may not be the only factor affecting feeding success. Described intraspecific diet differences that corresponded with morphological changes among benthic and pelagic feeding individuals. Additionally, Tobler (2008) found that resource use diverged among habitat types, and that jaw morphology was correlated with diet composition. These connections between fish morphology and resource use indicate the utility of adaptive phenotypic plasticity in response to pressures from resource availability.

The interannual comparison of streams revealed short‐term phenotypic changes to a deeper body morphology in the three streams studied in consecutive years. This was unexpected as we predicted shape to be stable within a stream population in the subsequent year, based on a previous morphometric analysis of temporal variation (Kern & Langerhans, 2018). Kern and Langerhans (2018) propose that though human activity may reduce biological diversity, it may also drive speciation by imposing new selective pressures. They report creek chub populations in urban streams exhibit a deep body shape in both modern‐day populations and museum samples from 50 years ago and identify lower flow in urban streams as a contributing factor. This contrasts with results presented here as creek chub populations were deeper bodied compared to the previous year. We attribute the interannual differences in shape to the dynamic quality of the stream systems in this region, as evident in the discharge data. The variation in flow over the course of the study period is relatively high and indicates the streams within this region experience drastic changes from 1 year to the next. Observationally, stream structure changed from 1 year to the next, with the location and depth of pools the most notable change and relevant to creek chub habitat. That creek chub populations in the region display varying morphologies reinforces the evolutionary strategy of a generalist species and its ability to utilize an adaptive phenotypic response to changing stream conditions, since environmental disturbances tend to select against specialist species (Buchi & Vuilleumier, 2014).

Phenotypic differences were observed in creek chub populations in consecutive years, which contrasted with our prediction to observe stable shape among populations over time. We identify two possible contributors to this phenomenon; either these streams in particular are changing rapidly between years, or morphometric analysis as a whole lacks an understanding of how quickly populations change. Most morphometric studies examine phenotypic responses in a single year, without accounting for temporal variation (Beachum et al., 2016; Haas et al., 2010; Lostrom et al., 2015; Meyers & Belk, 2014; Senay et al., 2015). When time is included as a predictor of shape change, it is typically done with museum specimens over tens of years, rather than on a short‐term scale (Franssen, 2011; Jacquemin & Pyron, 2016; Kern & Langerhans, 2018). It is also likely, given the documented flashy nature of these systems, that stream morphology and discharge changes in a short time period based on local precipitation events, which in turn pose additional selection pressures on the fish populations. Given this complication presented by variable stream conditions, it may be necessary to conduct more short‐term shape analyses to determine if it is common to document temporal differences.

Stream fish populations are exposed to variable conditions as a result of the nature of stream systems; river discharge and stream morphology are dynamic features which exert pressure on the organisms inhabiting these systems (Rhoads et al., 2003). In turn, these abiotic variables influence the entire organismal community which ultimately cascades to the fish populations. In some regions, like the Great Lakes, conditions may be even more volatile due in part to the growing influence of human activity within the watersheds which may increase the number of highly stressed ecosystems within the Great Lakes watersheds (Kovalenko et al., 2018; Mahdiyan et al., 2020). Though we determined that isolated populations of creek chub can display plastic responses in body shape, the complicated influence of various environmental factors and fluctuating river discharge and precipitation of these systems confirm the success of generalist fish species in dynamic systems. It should be no surprise that a generalist species is capable of adapting quickly to novel stream conditions, for this is the ultimate definition of a generalist species.

The management implications of this work are relevant for the reintroduction of a species to an area where it may have been extirpated due to an unsuitable habitat quality. We argue that these results show that a generalist species may be well suited to such a restoration, but that a more specialist species may need to be considered carefully for reintroduction. Though a generalist can adapt its body shape to accommodate changes to flow speed and its mouth shape to available resources, a specialist may not be able to adjust in the same way at a rapid pace, leaving it vulnerable to being outcompeted by other species. Rapid phenotypic plasticity in fish should be examined further to address the pace of change that can occur in fishes and determine if it is common or region‐specific. Additionally, given the flashy nature of these tributaries that may have contributed to interannual shape changes, creek chub populations in more stable streams could be examined to test these patterns.

## AUTHOR CONTRIBUTIONS

**Claire Hetzel:** Data curation (lead); formal analysis (equal); investigation (equal); writing – original draft (lead). **Patrick Forsythe:** Conceptualization (lead); formal analysis (equal); investigation (lead); resources (lead); writing – review and editing (lead).

## CONFLICT OF INTEREST STATEMENT

The authors declare no conflicts of interest.

## Supporting information


Appendix S1.

Figure S1.
Figure S2.
Figure S3.
Figure S4.
Figure S5.
Table S1.
Table S2.
Table S3.

## Data Availability

Raw landmark data for morphometric and multidimensional analyses are provided as supporting information. Further inquiries regarding environmental correlates or landscape metrics can be directed to the corresponding authors.
