# Supplementary material for: Phenotypic plasticity of a generalist fish species resident to lotic environments: Insights from the Great Lakes region
Source: Ecol Evol. 2023 Nov 20;13(11):e10715. doi: 10.1002/ece3.10715 (PMC10660395; doi:10.1002/ece3.10715)
Supplement: Supplementary file 2 — Figure S1. Figure S2. Figure S3. Figure S4. Figure S5. Table S1. Table S2. Table S3. [file ECE3-13-e10715-s002.docx]

**Supporting Information**

**Figure S1.** Average daily temperature for each study stream. Temperatures for the 2020 season were logged beginning August 14, while in 2021 loggers began recording August 18.


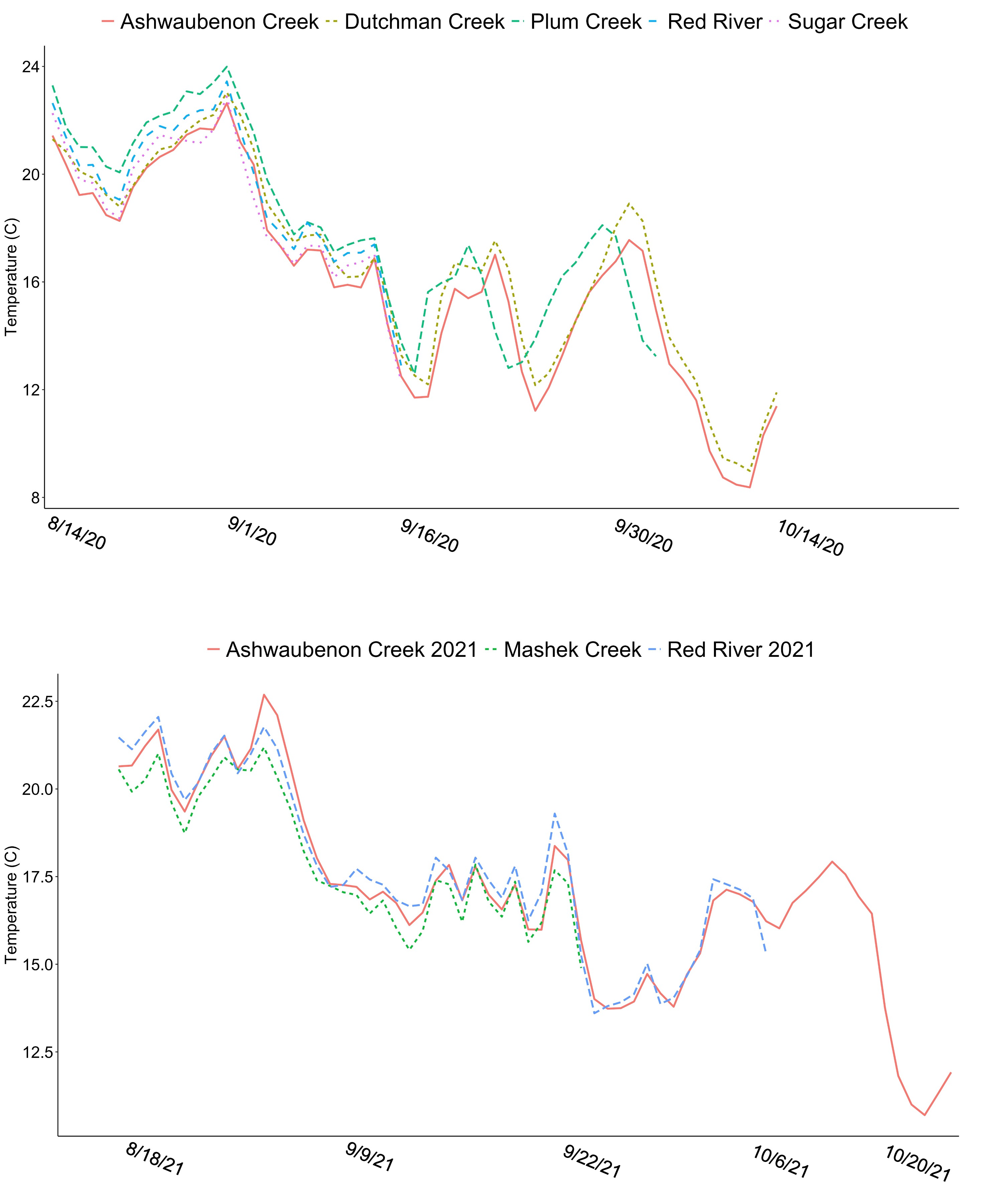


**Figure S2.** Stream discharge (m^3^/s) for Ashwaubenon Creek, Dutchman Creek, and Plum Creek prior to the sampling period and two months following sampling in 2020 and 2021, with dotted red lines indicated the sampling period. Data retrieved from USGS National Water Viewer.


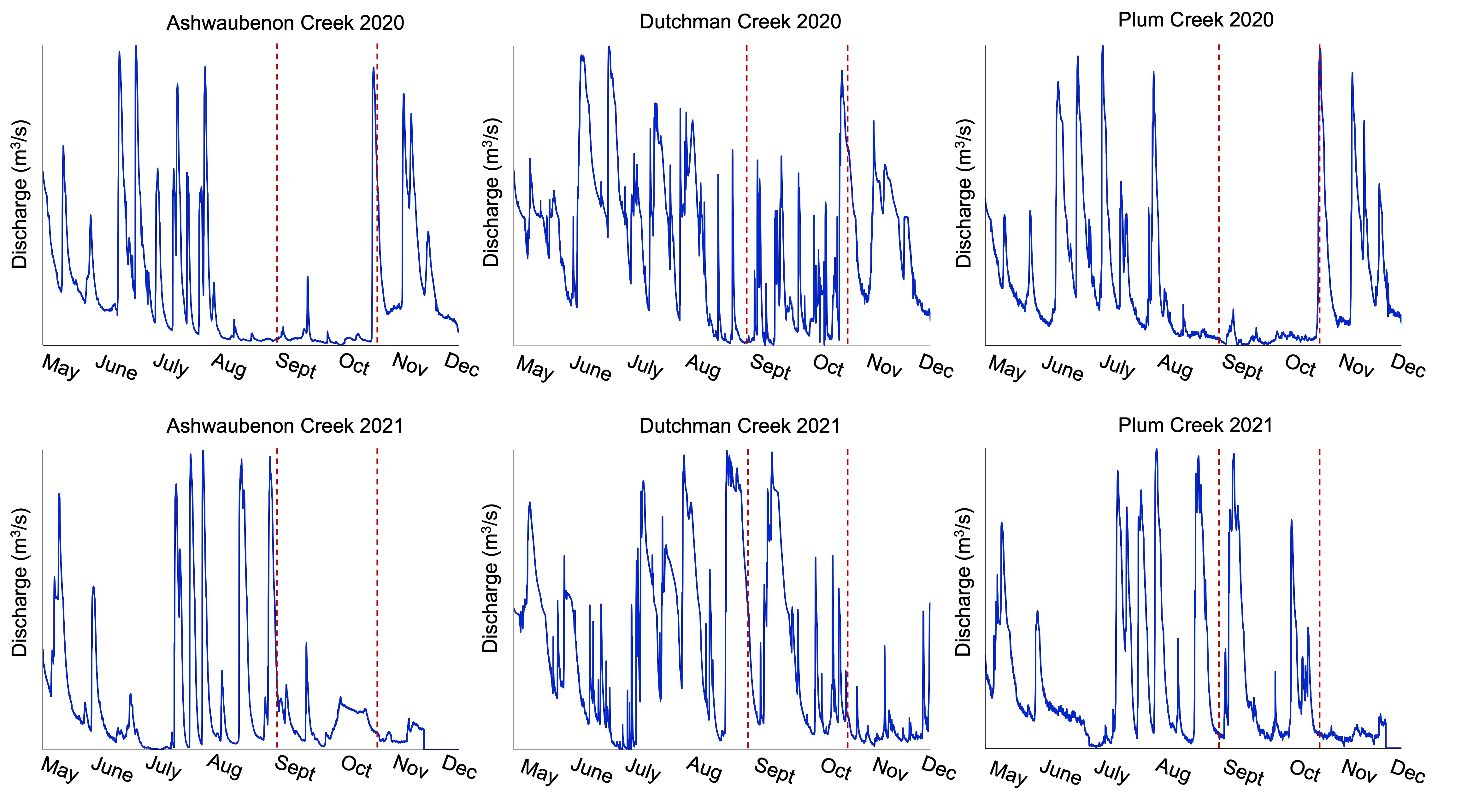


**Figure S3.** Example images of parasites discovered in gut content analysis.


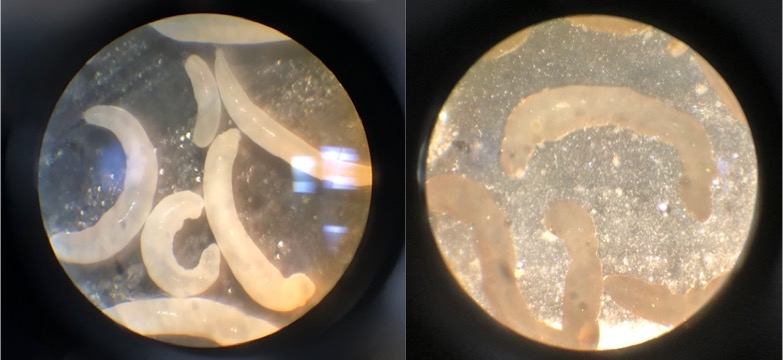


**Figure S4.** Full canonical variate plot of all sites, listed as stream abbreviation. **
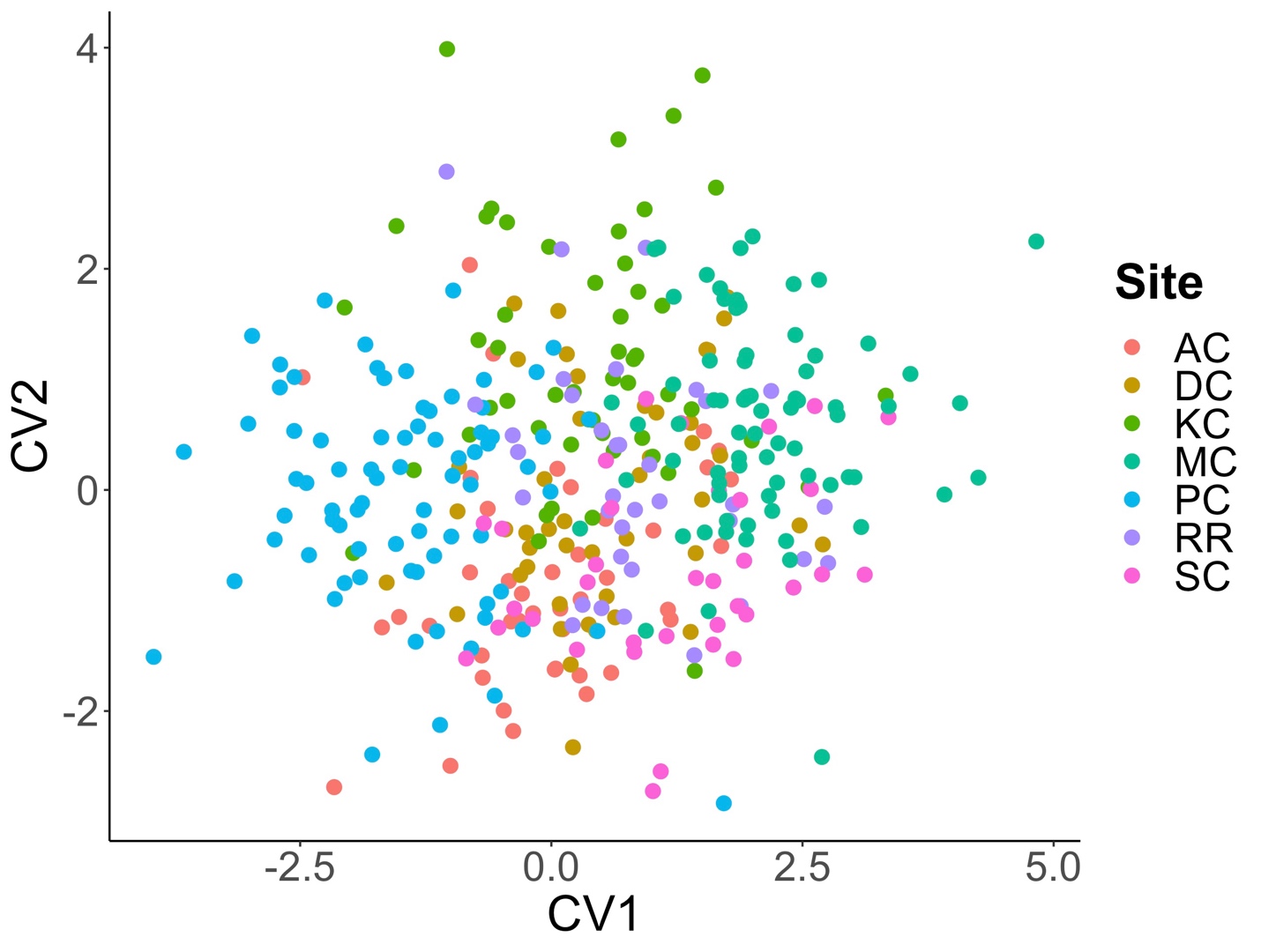
**

**Figure S5.** Average body shape for individual streams from the first sampling year. Average body shape for each stream population for the first sampling period. Wireframes are magnified by 3 to highlight shape differences.


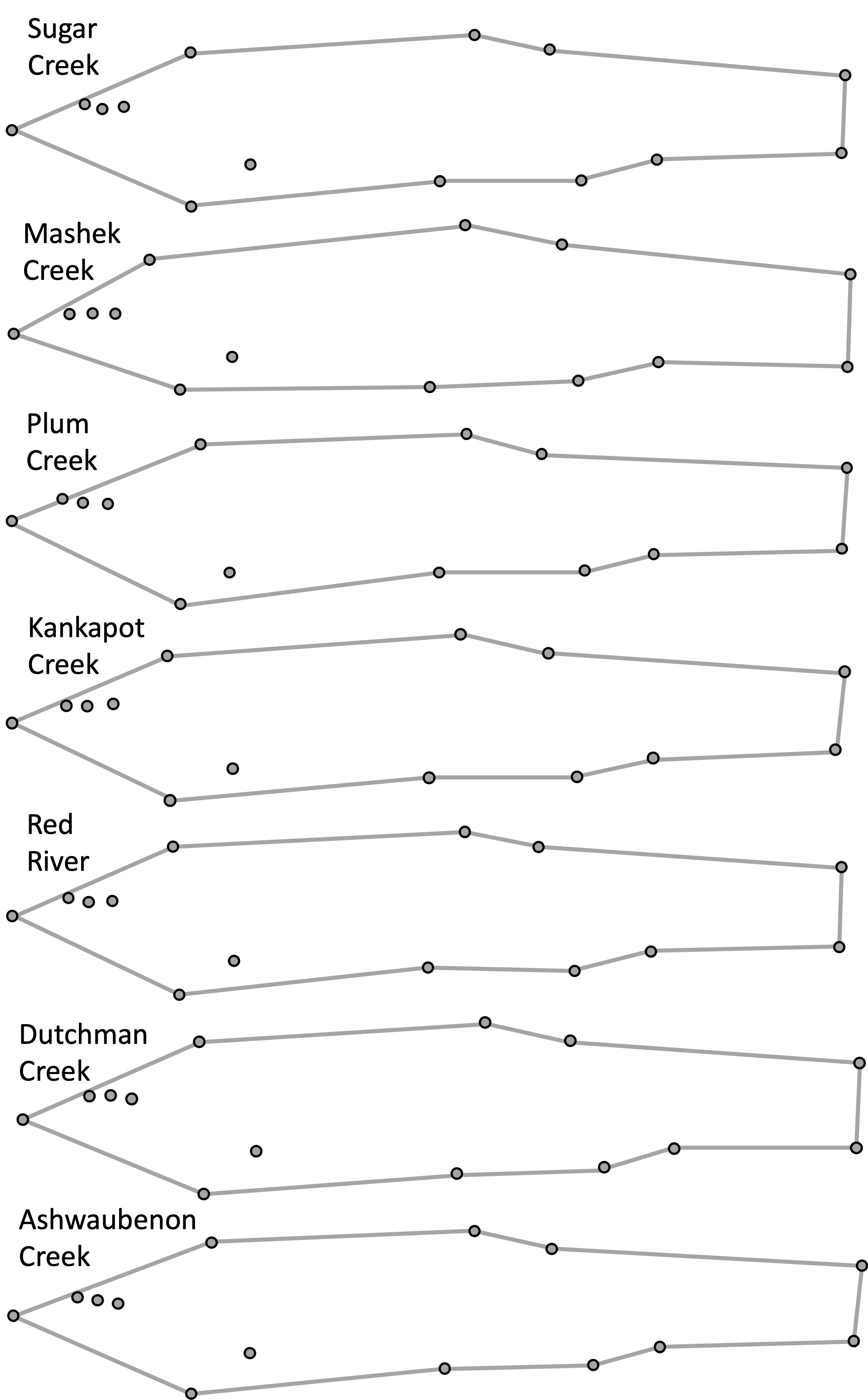


**Table S1.** Monthly average stream discharge (m^3^/s) for Ashwaubenon Creek (AC), Dutchman Creek (DC), and Plum Creek (PC) for 2020 and 2021, including the monthly average discharge for all streams.

|  | 2020 | | | |  | 2021 | | | |
| --- | --- | --- | --- | --- | --- | --- | --- | --- | --- |
|  | AC | DC | PC | Average |  | AC | DC | PC | Average |
| April | 0.206 | 0.465 | 0.182 | 0.2846 |  | 0.264 | 0.652 | 0.471 | 0.462 |
| May | 1.390 | 3.749 | 1.754 | 2.298 |  | 0.0730 | 0.244 | 0.133 | 0.150 |
| June | 0.308 | 0.766 | 1.386 | 0.820 |  | 0.273 | 0.634 | 0.993 | 0.633 |
| July | 0.300 | 0.566 | 0.496 | 0.454 |  | 0.967 | 1.1544 | 3.110 | 1.744 |
| August | 0.0157 | 0.0960 | 0.0250 | 0.0456 |  | 1.234 | 2.361 | 3.843 | 2.479 |
| September | 0.0180 | 0.146 | 0.0139 | 0.0595 |  | 0.0547 | 0.210 | 0.0662 | 0.110 |
| October | 0.392 | 0.877 | 1.333 | 0.867 |  | 0.0472 | 0.0900 | 0.289 | 0.142 |
| November | 0.360 | 0.569 | 0.722 | 0.550 |  | 0.0214 | 0.0348 | 0.0377 | 0.0313 |
| December | 0.0560 | 0.0928 | 0.0703 | 0.0730 |  | 0 (ice) | 0.0763 | 0.0220 | 0.0328 |

**Table S2.** Relative importance of prey items (as percent of total) for each stream.

| Prey Item | Sugar Creek | Red River | Kankapot Creek | Plum Creek | Ashwaubenon Creek | Dutchman Creek | Mashek Creek |
| --- | --- | --- | --- | --- | --- | --- | --- |
| Plant material | 23.76 | 0 | 6.63 | 2.98 | 13.44 | 0 | 0 |
| Unidentified insect | 18.20 | 22.92 | 3.19 | 6.00 | 3.29 | 72.55 | 26.29 |
| Unidentifiable | 5.46 | 0.17 | 54.48 | 85.76 | 1.87 | 1.37 | 25.58 |
| Parasites | 38.88 | 72.01 | 19.30 | 0.54 | 1.19 | 0.80 | 34.87 |
| Detritus | 13.44 | 2.13 | 9.25 | 3.65 | 27.95 | 4.53 | 0 |
| Terrestrial insect | 0.13 | 0.18 | 2.40 | 0 | 0.92 | 4.80 | 8.03 |
| Worm | 0.13 | 0 | 0 | 0 | 0.40 | 1.04 | 0 |
| Wing fragment | 0 | 1.64 | 0 | 1.05 | 0.40 | 1.94 | 0.42 |
| Hemiptera | 0 | 0 | 0 | 0 | 1.33 | 11.24 | 0.37 |
| Coleoptera | 0 | 0.47 | 0 | 0 | 15.62 | 1.74 | 0.51 |
| Trichoptera | 0 | 0 | 4.75 | 0 | 33.59 | 0 | 0 |
| Bone fragment | 0 | 0.17 | 0 | 0 | 0 | 0 | 0.57 |
| Chironomid | 0 | 0.32 | 0 | 0 | 0 | 0 | 0.57 |
| Fish | 0 | 0 | 0 | 0 | 0 | 0 | 0 |
| Snail | 0 | 0 | 0 | 0 | 0 | 0 | 0.55 |
| Ephemeroptera | 0 | 0 | 0 | 0 | 0 | 0 | 0.51 |
| Megaloptera | 0 | 0 | 0 | 0 | 0 | 0 | 0.37 |
| Odonata | 0 | 0 | 0 | 0 | 0 | 0 | 0.79 |
| Amphipod | 0 | 0 | 0 | 0 | 0 | 0 | 0.57 |
| Crayfish | 0 | 0 | 0 | 0 | 0 | 0 | 0 |

**Table S3.** Full regression results for the first two relative warps, with significant results in bold.

| Model term | Predictor variable | Coefficients | *F* | *df* | *p* | *r^2^* |
| --- | --- | --- | --- | --- | --- | --- |
| RW 1 | Centroid size | 0.03404 | 0.0307 | 5 | 0.8677 | -0.1927 |
|  | Total length | 2.971e-04 | 1.183 | 5 | 0.3263 | 0.0297 |
|  | Average temperature | 0.0023062 | 0.4322 | 4 | 0.5468 | -0.1281 |
|  | Watershed size | -1.364e-04 | 1.908 | 5 | 0.2258 | 0.1314 |
|  | % Developed land cover | 3.704e-04 | 3.082 | 5 | 0.1395 | 0.2576 |
|  | % Agriculture land cover | 3.455e-04 | 0.8618 | 5 | 0.3958 | -0.0236 |
|  | Fish community diversity | -0.01439 | 0.4196 | 5 | 0.5457 | -0.1071 |
|  | Creek chub dominance (%) | 0.0001424 | 0.5097 | 5 | 0.5072 | -0.0890 |
|  | Prey diversity | -0.012446 | 0.4222 | 5 | 0.5445 | -0.1066 |
| RW 2 | Centroid size | 0.08667 | 0.6177 | 5 | 0.4675 | -0.0681 |
|  | Total length | 6.129e-05 | 0.1157 | 5 | 0.7475 | -0.1729 |
|  | Average temperature | 0.0004270 | 0.0351 | 4 | 0.8606 | -0.2391 |
|  | Watershed size | 5.844e-05 | 0.8194 | 5 | 0.4069 | -0.0310 |
|  | % Developed land cover | -1.686e-04 | 1.408 | 5 | 0.2888 | 0.0636 |
|  | % Agriculture land cover | -4.191e-04 | 7.529 | 5 | **0.0406** | 0.5211 |
|  | Fish community diversity | 0.010440 | 0.6387 | 5 | 0.4604 | -0.0641 |
|  | Creek chub dominance (%) | -0.0001392 | 1.627 | 5 | 0.2581 | 0.0946 |
|  | Prey diversity | -0.020225 | 6.663 | 5 | **0.0494** | 0.4856 |
